# Crosslinked Chitosan Films Supplemented with *Randia* sp. Fruit Extract

**DOI:** 10.3390/polym15122724

**Published:** 2023-06-18

**Authors:** Felipe López-Saucedo, Leticia Buendía-González, Héctor Magaña, Guadalupe Gabriel Flores-Rojas, Emilio Bucio

**Affiliations:** 1 Facultad de Ciencias, Campus El Cerrillo Piedras Blancas, Universidad Autónoma del Estado de México, Carretera Toluca-Ixtlahuaca Km 15.5, Toluca 50200, Mexico; 2 Departamento de Química de Radiaciones y Radioquímica, Instituto de Ciencias Nucleares, Universidad Nacional Autónoma de México, Circuito Exterior, Ciudad Universitaria, Mexico City 04510, Mexico; 3 Facultad de Ciencias Químicas e Ingeniería, Universidad Autónoma de Baja California, Calzada Universidad 14418, Parque Industrial Internacional Tijuana, Tijuana 22390, Mexico

**Keywords:** biomaterial, chitosan, crosslinking, hydrogel, natural product, *Randia capitata*

## Abstract

This work proposes the development of a polymer film made up of affordable components for its use as a healthcare material. Chitosan, itaconic acid, and *Randia capitata* fruit extract (Mexican variation) are the unique ingredients of this biomaterial prospect. Chitosan (from crustacean chitin) is crosslinked with itaconic acid, and in situ added *R. capitata* fruit extract in a one-pot reaction carried out in water as the sole solvent. Structurally, the film formed is an ionically crosslinked composite characterized by IR spectroscopy and thermal analysis (DSC and TGA); cell viability was also performed in vitro using fibroblasts BALB/3T3. Dry and swollen films were analyzed to determine affinity and stability in water. This chitosan-based hydrogel is designed as a wound dressing due to the combined properties of the chitosan with *R. capitata* fruit extract, which has potential as bioactive material due to its properties in epithelial regeneration.

## 1. Introduction

In recent years, the trend of using natural materials has become popular. Preference for materials obtained from plants, animals, or microorganisms over synthetics is diverse; one benefit is that once the life cycle is closed, the residues can be easily reincorporated into the environment as new mineral nutrients [1], unlike synthetic polymer materials that require careful residue management once disposed [2]; unfortunately, however, synthetic polymers usually turn into pollutant agents due to poor treatment. For example, waste plastics cause serious damage to soils [3] and effluents such as rivers, lakes, and seas [4]. A way to help stop the disproportionate increase of human waste is promoting the use of biodegradable materials to reincorporate simpler compounds rich in C, N, and O into the environment to be used as nutrients for plants and microorganisms [5].

Chitosan is a polymer of natural origin obtained by deacetylation of chitin, which is abundantly found in the exoskeletons of arthropods and the cell wall of some fungi [6,7]. Chitosan contains −OH groups that allow the formation of intermolecular hydrogen bonds; it also has a −NH_2_ group, which can react with electrophiles such as carboxylic acids −COOH to form covalent bonds in a condensation (forming amides), or ionic bonds (forming carboxylate-ammonium ions) [8]. This polysaccharide is quite thermally stable and is structured of linear chains, but can be easily crosslinked [9], so it is possible to obtain higher molecular weight polymers to form films or hydrogel networks [10]. The use of chitosan is convenient for sanitary dressing, nanoparticle loading [11], and drug loading/delivery systems [7,12] due to its high hydrophilicity. Traditionally, the crosslinking of chitosan hass been carried out in acetic acid [13,14], with different crosslinking agents [15,16,17], and even using ionic liquids as solvent [18]. Recently, the crosslinking of chitosan and itaconic acid has been reported with promising results [8,19], where the versatility of this diacid functioning as a crosslinking agent is highlighted.

*Randia* sp. is a fruity arboreal plant belonging to the Rubiaceae family that is found around the world [20], including Mexico, where it is known by different names, including “crucecita”, “cruceta”, and “crucillo”. In the past, the fruit of this plant was used in traditional medicine, although recently in this century some varieties of *Randia*, such as *R. aculeata* [21], *R. echinocarpa* [22,23], and *R. dumetorum* [24,25] have attracted the attention of organic chemistry and pharmacology, since there is evidence of their bioactivity. Thus, incorporating *Randia* sp. extracts into biomedical devices and disposables could be an eco-friendly alternative for healing purposes.

This paper puts forward a film containing only three natural-based ingredients chitosan (polymer), itaconic acid (crosslinking agent), and *R. capitata* fruit extract (bioactive compound). This film was synthesized in water as the sole solvent and under catalyst-free conditions, which is our proposed method. The goal is to get an absorbable, biodegradable, and biocompatible film as a potential wound dressing [26].

## 2. Materials and Methods

### 2.1. Reagents and Solvents

Chitosan (75–85% deacetylated, viscosity 200–800 cP, 1 wt.%, in acetic acid (1% vol.), at 25 °C, CAS 9012-76-4) and itaconic acid (99%) were obtained from Sigma Aldrich (South 2nd, Saint Louis, MO, USA). Dry fruits of *Randia capitata* (*R. capitata*) were harvested from trees located in Veracruz, Mexico. Double distilled water was used for the reaction and analysis, and ethanol reagent grade from Sigma Aldrich (South 2nd, Saint Louis, MO, USA) was used to isolate the *Randia* extract.

### 2.2. Randia Extract

Complete dry fruit *R. capitata* was pulverized with a mortar. Then, 1 g of the powder was placed into a flask (100 mL capacity), dissolved in 20 mL of a 1:1 water/ethanol mixture, and sonicated for 30 min at room temperature. Separation of solid and liquid phases was performed in a vacuum line system with a funnel. Whatman filter paper (number 3) and fiberglass were used to retain most of solid particles and to allow a faster filtration. The liquid phase obtained was reserved inside a ball flask, and subsequently the solution was concentrated under reduced pressure to isolate the *R. capitata* extract (a semi-solid), which was stored at 4 °C.

### 2.3. Synthesis of Chitosan/Itaconic Acid Film

**Figure 1:**
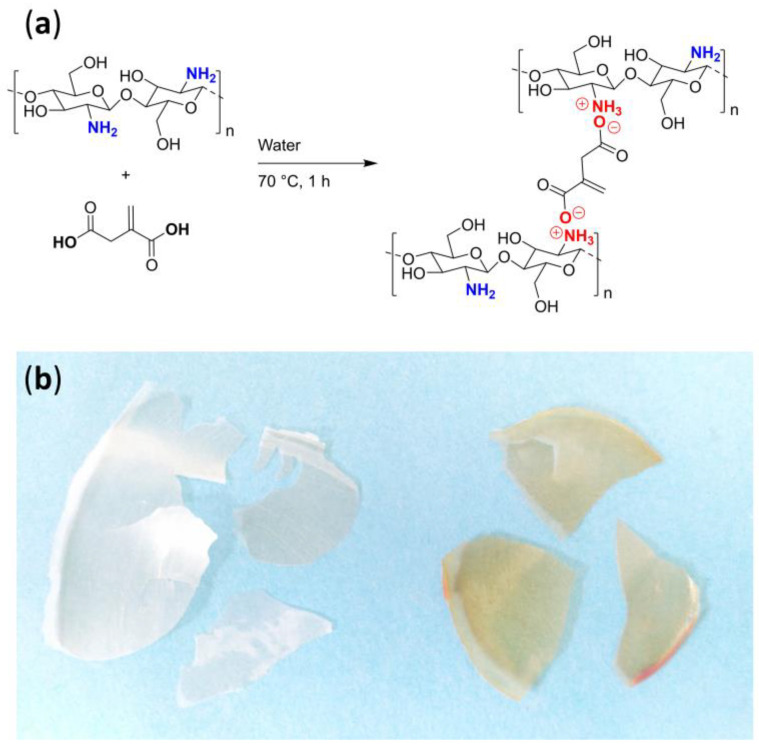
(**a**) Scheme of synthesis of ionic crosslinking of chitosan and itaconic acid. (**b**) Side by side chitosan/itaconic acid film (**left**) and chitosan/itaconic acid/*Randia* film (**right**).

Chitosan/itaconic acid film (Ch/It) was prepared mixing 100 mg of chitosan and 42 mg of itaconic acid in 5 mL of water inside a beaker of 100 mL capacity. Composition was based on stoichiometry of chitosan units and molecular weight of itaconic acid (Figure 1a). This mixture was kept under stirring for 60 min at 70 °C. Once the reaction time was over, the magnetic stirrer was removed, and the solution (of viscous appearance) remained inside the beaker at room temperature for 72 h, to allow slow evaporation of water. The dry film was carefully taken out the bottom of beaker and stored into a vacuum desiccator chamber at room temperature until use (Figure 1b left).

### 2.4. Synthesis of the Chitosan/Itaconic Acid/Randia Film

Chitosan/itaconic acid/*Randia* film (Ch/It/Ran) was synthesized, weighing 100 mg of chitosan and 42 mg of itaconic acid in 5 mL of water. This mixture was stirred for 60 min at 70 °C and cooled until room temperature. Then, 3 mL of an aqueous solution containing 40 mg of *Randia* extract was added. This amount of fruit extract was chosen because a homogeneous film was obtained, as well as the high cell viability observed (see Section 3.6). The film was dried at room temperature for 72 h and stored inside a desiccator at room temperature until use (Figure 1b right).

### 2.5. Limit Swelling

Small pieces of Ch/It or Ch/It/Ran films (between 2 and 4 mg) were weighed and placed inside small aluminum pans. Later, distilled water at room temperature was added until the sample got submerged. Swelling measurements were chronometered and monitored for up to 6 h. The swollen film was taken out of the water in between each weighing, the excess water was absorbed with a paper wipe to weigh the sample in the balance, and according to Equation (1) the *Swelling (%)* was calculated.

*Swelling (%) = [ws − wd/wd] × 100* (1)

where *ws* is the weight of swollen film (hydrogel) and *wd* is the weight of dry film.

### 2.6. Cell Viability 

The fibroblast cell line BALB/3T3 (mouse) was used to test the cell viability of different samples Ch/It, Ch/It/Ran, *Randia* complete fruit, and *Randia* fruit extract in in vitro conditions. First, an indirect method was performed, in which 20 mg of each sample was suspended in 2 mL of DMEM (Dulbecco’s Modified Eagle Medium) for 24 h. Subsequently, 50 µL of this medium was placed in contact with cell medium in 96-well plates. Each well contained 30,000 cells mL^−1^ in DMEM medium complemented with fetal bovine serum medium, streptomycin solution, and gentamicin. Incubation was for 24 h at 5% CO_2_ and 37 °C. Subsequently, 3-(4,5-dimethylthiazol-2-yl)-2,5-diphenyltetrazolium bromide (MTT) kit reagent (Roche, Switzerland) was placed in each well to determine the colorimetric cell viability. The absorbance of samples and controls was recorded at a wavelength of 620 nm. Cell viability was determined using Equation (2). Results were performed in triplicate, collected, and statistically analyzed.

*Cytocompatibility (%) = [A Sam)/[A Con] × 100* (2)

where *A Sam* is the absorbance of the sample, and *A Con* corresponds to absorbance of the control recorded at 620 nm.

### 2.7. Instrumental

#### 2.7.1. UV-Vis Spectrophotometry 

A Multiskan FC spectrophotometer (Thermo Scientific, Waltham, MA, USA) was used to determine the absorbance at a wavelength of 620 nm. Cell viability was performed in 96-well plates. The experiments were performed in triplicate.

#### 2.7.2. Infrared Spectroscopy

A Perkin-Elmer Spectrum 100 (PerkinElmer, Inc., Waltham, MA, USA) equipped with ATR was used to analyze all samples. First, a background scan was run, and then the dry sample was analyzed with 16 scans in the wavelength range from 4000 to 650 cm^−1^.

#### 2.7.3. Differential Scanning Calorimetry (DSC)

Differential scanning calorimetry was carried out in a Differential Scanning Calorimeter 2010 (TA Instruments, New Castle, DE, USA). Around 10 mg of a dry sample was weighed, encapsulated in an aluminum pan, and analyzed from 25 to 250 °C in a nitrogen atmosphere at a heating rate of 10 °C min^−1^.

#### 2.7.4. Thermogravimetric Analysis (TGA)

Thermogravimetric analysis was performed using a TGA Q50 (TA Instruments, New Castle, DE, USA). Then, 5 to 10 mg of a sample previously dried into a vacuum oven at 60 °C was weighed. Each sample was placed in a platinum tray, and the analysis was performed at a heating rate of 10 °C min^−1^ up to 800 °C under nitrogen atmosphere.

#### 2.7.5. Contact Angle

Samples of Ch/It and Ch/It/Ran were selected and placed between two glasses and pressed for 2 days at room temperature to get flattened surfaces. For this technique, a Kruss DSA 100 droplet analyzer (Hamburg, Germany) was used. The native software was used to determine the angles formed between the film surface and the water microdroplet. Angles were recorded at 0, 1, and 2 min; each experiment was performed in triplicate.

#### 2.7.6. Direct Analysis in Real-Time Mass Spectrometry (DART-MS)

The mass spectrometer instrument was a JEOL AccuTOF: JMS-T100LC with ionization mode DART^+^.

## 3. Results and Analysis

### 3.1. Mass Spectrometry of Randia Extract

Regarding the composition of the ethanol/water *Randia* extract, it was found in the literature that alcoholic *Randia* extracts contain mainly nitrogen-free compounds, particularly phenols and flavonoids, characterized by ferric chloride and Constantinescu assays, respectively. In said investigation, the total composition of *Randia laevigata* fruit alcoholic extract was 57% humidity, 3% ashes, 3.31% protein, and 37.75% nitrogen-free molecules [27].

**Figure 2:**
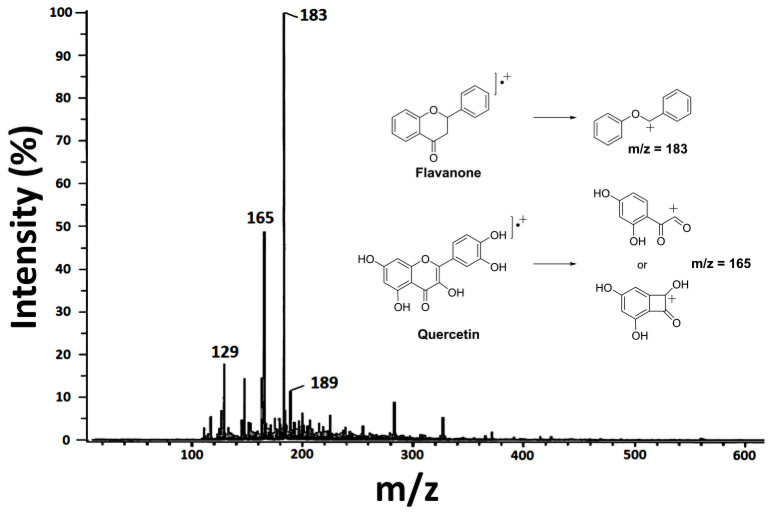
DART+ mass spectrometry of *Randia* extract and proposed fragmentation of two flavonoids corresponding to the main signals detected.

Following the reports by Santos-Cervantes et al. [28] and Cruz-Silva et al. [29], who reported the abundance of flavonoids in alcohol *Randia* extracts, in this investigation, with the ethanol/water *Randia capitata* extract, the existence of flavonoids was confirmed, where the presence of flavanone and quercetin is likely. Since in the DART^+^ spectrum (Figure 2), the most intense peaks correspond to these compounds; firstly, the base peak m/z = 183 may be attributed to the fragmentation of flavanone in the alpha position to the carbonyl, while the second most intense peak at m/z = 165 possibly corresponds to the quercetin fragment formed by a rearrangement during ionization [30].

### 3.2. Spectroscopic Analysis

**Figure 3:**
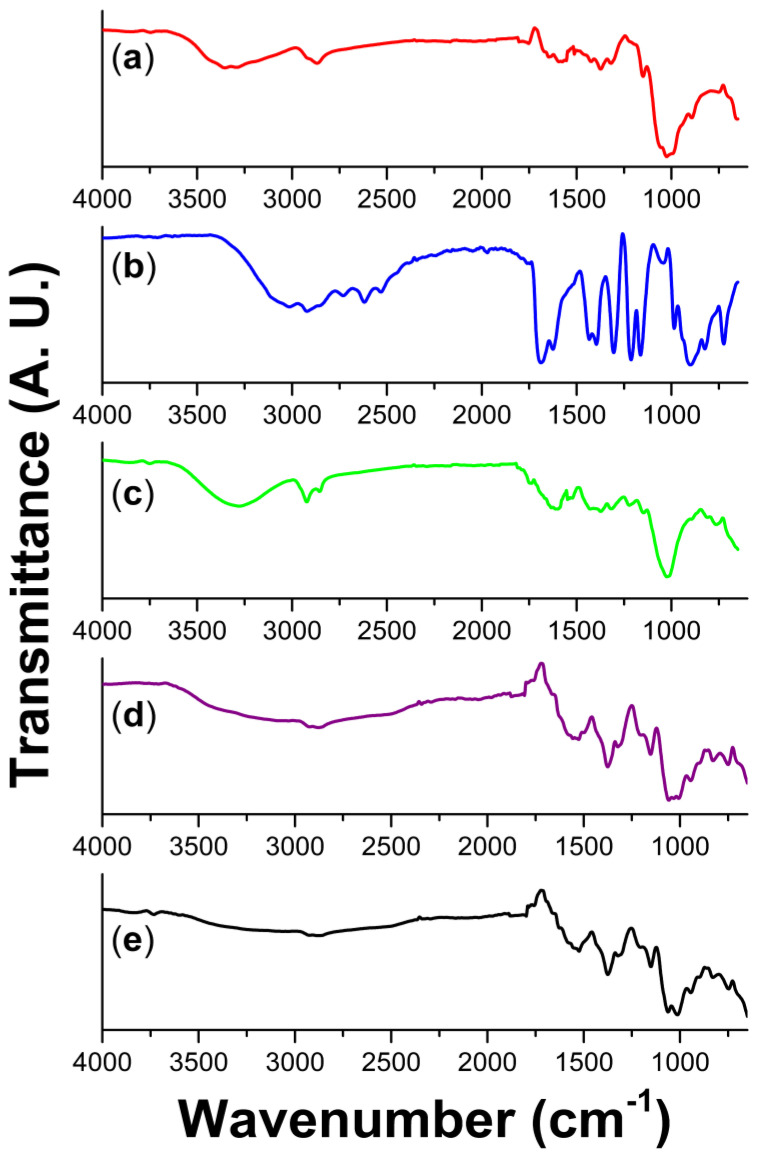
FTIR−ATR spectra of (**a**) chitosan, (**b**) itaconic acid, (**c**) *Randia* extract, (**d**) Ch/It, and (**e**) Ch/It/Ran.

IR-ATR spectroscopy was carried out in dry samples to avoid signals of absorbed water from moisture. The starting compounds chitosan, itaconic acid, and *Randia* extract share functional groups in common, but there are certain differences that allow their characterization (Figure 3) before and after crosslinking reaction (in the composite films). First, in the chitosan spectrum, the O-H stretching band is distinguished, which is wide and appears above 3000 cm^−1^; in this zone, two small overlapped peaks at 3354 and 3296 cm^−1^ may correspond to N-H stretching of amines; and at 2868 cm^−1^, the C-H stretching band is observed; at 1575 cm^−1^ appears the N-H bending; while the symmetric methyl bending signal is observed at 1373 cm^−1^; finally, at 1145 and 1025 cm^−1^, the asymmetric and symmetric stretching of C-O are observed [31]. In the spectrum of itaconic acid, besides the bands of O-H and C-H stretching, the C=O of carbonyl from the diacid is observed at 1682 cm^−1^; the alkene band of C=C appears at 1623 cm^−1^, which is particularly strong; methyl bending at 1393 cm^−1^ is distinguished; and finally, the C-O stretching bands appear at 1215 and 1164 cm^−1^. The third spectrum of *Randia* extract, due to its natural origin, displayed similar bands to those found in natural products (plants and fruits) [32]; despite that *Randia* extract is a mixture, it was possible to identify some bands, for example, at 3291 cm^−1^ the stretching of O-H, which is observed in carbohydrates, polysaccharides, aliphatic alcohols, and also in phenols [23]. Additionally, in the zone of 1600–1000 cm^−1^, the secondary signals of C-H, O-H, and C-C confirmed the bonds found in the mentioned natural compounds (Table 1). 

Regarding FTIR of films, in addition to functional groups already described, there was evidence of the crosslinking reaction, with forming the ionic bond R-COO^−^ R-NH3^+^ from carboxyl (from itaconic acid) and the amine (from chitosan). In both infrared spectra of Ch/It and Ch/It/Ran, the carboxylates are observed, although in the Ch/It film it is easier to identify the carboxylate anion R-(O=C-O^−^), since the band corresponding to the asymmetric stretching is observed at 1528 cm^−1^ and the symmetric stretching band is also observed at 1375 cm^−1^ [33], which overlaps with the methyl bending and is intensified for this reason. Although the O-H stretching band should decrease, it was not the case, since the band appeared more intense and broad, due to humidity and/or residual water from the reaction. Moreover, there was no evidence of carbonyl of amides. The carbonyl band broadened, and stronger bands are a consequence of a higher molecular weight; in other words, the chitosan/itaconic acid is crosslinked through the formation of ionic bonds of ammonium-carboxylate [9]. On the other hand, in the Ch/It/Ran film, the O-H bands are also observed, and the band at 1524 cm^−1^ of carboxylate anion is observed; while the intense band at 1375 cm^−1^ is attributed to two types of vibrations, these are carboxylate symmetric stretching and methyl bending; finally, the group of bands at 1151, 1061, and 1013 cm^−1^ confirmed the C-O stretching of alcohols and phenols from flavonoids [34], as well as complex carbohydrates [22] and polyglucosamine [35].

Summarizing, FTIR spectra indicated that the Ch/It and Ch/It/Ran films formed ionic crosslinked structures through the carboxylic and amino groups, and conformed intermolecular hydrogen bonds; this is confirmed, since the film bands become more intense and they broaden when the molecular weight of chitosan is increased, as in hydrogels [36]. 

### 3.3. Mechanism of Crosslinking

Regarding the crosslinking reaction mechanism, Sirviö et al. found that during the crosslinking of itaconic acid and chitosan in the first step, the addition between the alkene group of itaconic acid and the amine of chitosan takes place. In the second step, the amine intermediate subsequently reacts with the carboxyl group of itaconic acid, resulting in a 5-membered cyclic amide (or substituted γ-lactam), where reaction conditions at reflux temperature and reaction time of 24 h were reported [19].

Other works also shed some light concerning this type of reaction. For example, Qu et al. reported the polymerization of ethanolamine with itaconic acid in N_2_ atmosphere, heating at 180 °C, and using a catalyst [37], which are extreme reaction conditions compared to those reported in this manuscript. On the other hand, Milosavljevic et al. (like us) proposed that ionic crosslinking is the primary mechanism, since the formation of carboxylate and ammonium species is favored in an aqueous reaction medium [8,38]. While in an independent research, a similar ionic crosslinking mechanism was reported for chitosan with oxalic acid [39]. Finally, according to Gabriele et al., it is indicated that during crosslinking of chitosan and succinic acid to form membranes, both, the formation of covalent and ionic bonds occurred [40]. Once again, the reaction conditions determine the path of bonding during the reaction.

### 3.4. Hydrophilicity: Contact Angle and Swelling

Both Ch/It and Ch/It/Ran films were subjected to two different tests to determine the degree of hydrophilicity; these tests were the contact angle per drop of water and swelling by immersion in water. The experiments were carried out at room temperature with double distilled water, with the following results.

**Figure 4:**
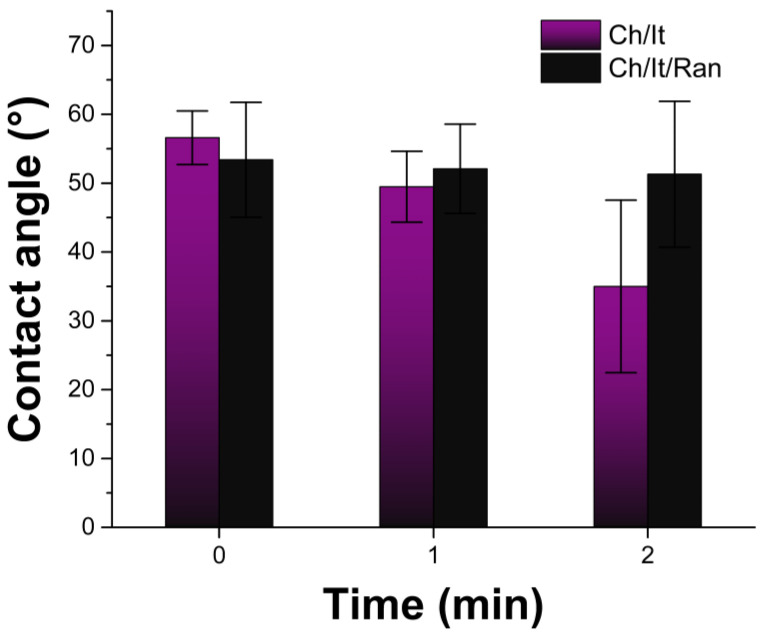
Contact angle of Ch/It and Ch/It/Ran measures between 0–2 min.

The contact angle was measured at short times because at longer times the surface of the film is deformed, and recording is not correct. Both films showed contact angles under 90° at time = 0, which means that surfaces wet quickly and are hydrophilic; in addition, the films increase in size when the experiment is over, which is a first indication of physisorption phenomena [41]. Quantitatively, the Ch/It angles range from 56 ± 3.9° to 35 ± 12.5°, while for the Ch/It/Ran film, the angles found were between 53.4 ± 8.3° and 51.3 ± 10.6° (Figure 4), which means that the Ch/It film is more hydrophilic and the drop of water adheres to the surface faster; it is important to notice that the error bars are larger at 2 min, which is attributed to the difficulty of measuring the angle due to deformations on the surface caused by the water droplet absorbed.

**Figure 5:**
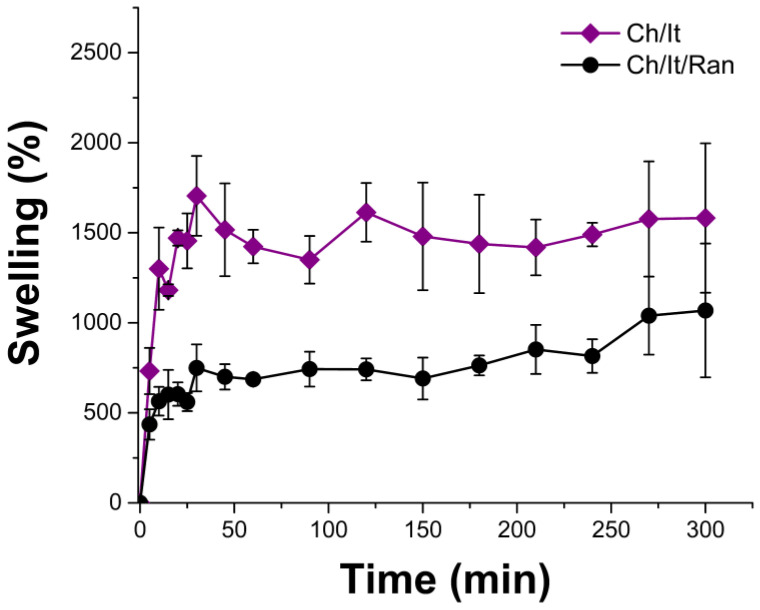
Percentage of water uptake of samples in distilled water.

Swelling capacity of films was measured by gravimetry (Figure 5). Ch/It film swelled the most (1582 ± 415%), and even reached the fastest maximum swelling in the first minutes, while the Ch/It/Ran film showed a curve with a less pronounced slope in the first minutes and with a lower swelling percentage (1069 ± 372%). Despite the differences in the swelling percentages, in both films this physicochemical behavior is like that found in hydrogels [7,42,43]. Because of this affinity for water, it is assumed that the interactions between the water in the medium and the films is not limited to the surface; water molecules most likely penetrated between the crosslinked polymer chains forming hydrogen bonds, being that this water retained and facilitated a fast saturation (swelling limit) [44]. The wettability and subsequent swelling of the films did not cause their destruction by infinite swelling; this is possible because the crosslinking of the films stops the swelling, since the ionic bond between the carboxylate and the amino groups must be stronger [45] than the hydrogen bond with the surrounding water [46]. However, at a macroscopic level, the film can be broken by rude mechanical action, so films must be carefully handled to keep its integrity.

### 3.5. Thermal Analysis

Reagents (raw materials) and products were subjected to two types of thermal analysis, DSC and TGA. In general, it was noticed that the thermal properties of the analyzed films versus reagents have a certain relationship, although it is evident that the films are not just a mixture; the formation of ionic bonds between chitosan and itaconic acid through a crosslinking reaction is inferred, since the transitions found in the films correspond to a material with new characteristics.

**Figure 6:**
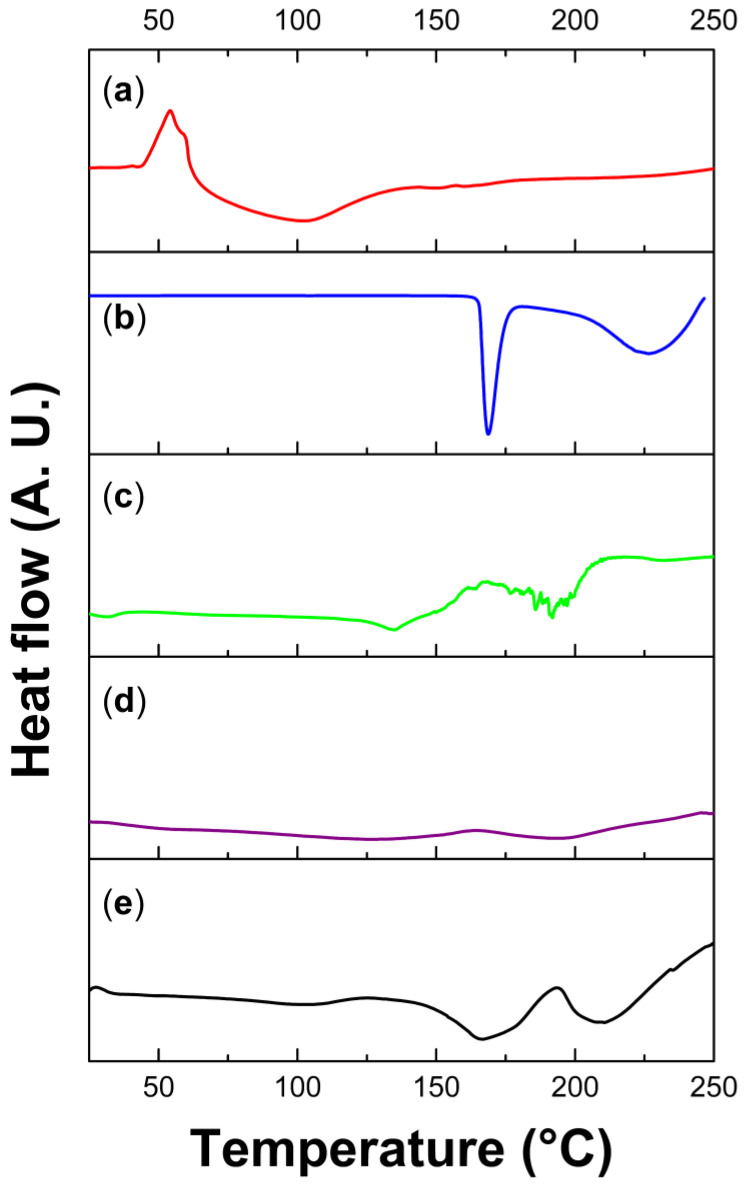
DSC thermograms of samples run under nitrogen atmosphere, up to 250 °C, and heating rate at 10 °C min^−1^. (**a**) chitosan; (**b**) itaconic acid; (**c**) *Randia* extract; (**d**) Ch/It; (**e**) Ch/It/Ran.

In the analyzed DSC thermograms, mainly endothermic transitions are observed (Figure 6); in the DSC of chitosan, the largest signal is a very broad and undefined endothermic transition that reaches the maximum energy change at 101.48 °C and is attributed to water loss [6]. For itaconic acid thermogram, a narrow signal that corresponds to the melting temperature (Tm) is observed; this type of signal appears in crystalline organic compounds of high purity. In the thermogram of the *Randia* extract, an endothermic transition of intermediate intensity is observed at 135 °C; in addition, several peaks are observed between 172 and 208 °C (with a midpoint at 191 °C) (Table 2), and these signals are attributed to the large number of organic compounds present in the extract with Tm in this temperature range. Finally, in the DSC thermograms of the Ch/It film, it is not possible to clearly observe transitions, and only a small endothermic transition is observed at 128 °C, which can be attributed to water loss; this lack of signals is indicative of films with a mainly amorphous structure. In the thermogram of the Ch/It/Ran film, two small (but recognizable) endothermic transitions appear at 167 and 210 °C that can be attributed to domains of itaconic acid and *Randia* extract, due to the proximity of the Tm of these compounds.

**Figure 7:**
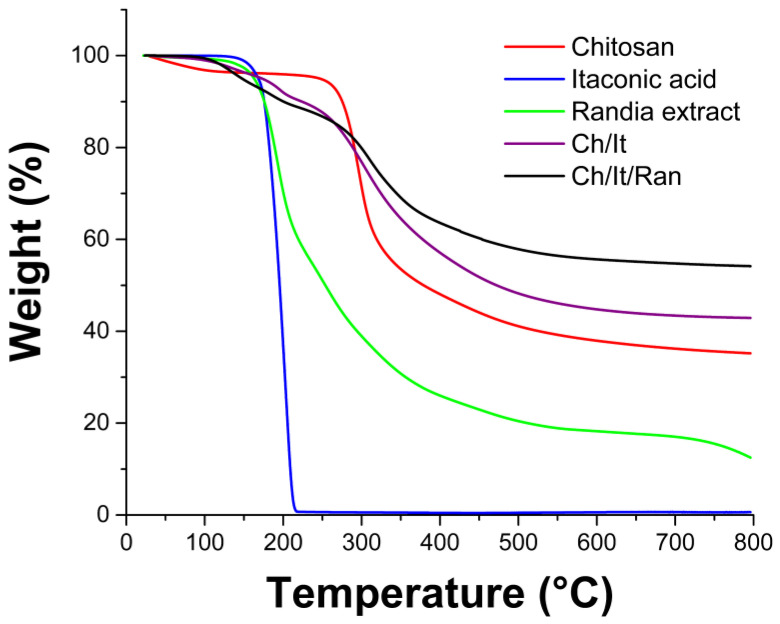
TGA thermograms of samples run at conditions of nitrogen atmosphere, up to 800 °C, and heating rate at 10 °C min^−1^.

The information collected from TGA thermograms completed the thermal analysis (Figure 7). It was found that 10% weight loss temperature was higher for chitosan, followed by Ch/It/ and Ch/It/Ran, while the lowest temperatures were for *Randia* extract and itaconic acid, respectively. This trend was also observed in the decomposition temperatures (Td). It should be noted that chitosan and itaconic acid show a single Td, while the Td of the *Randia* extract and the Ch/It/ and Ch/It/Ran films show multi-stage decompositions, due to the components of these mixtures having different resistance to heating. Respecting the analysis at 800 °C, the Ch/It/Ran film was the one that most resisted heating with 54.2% of residue mass, due to the crosslinking with itaconic acid and a structural reinforcement with the *Randia* extract; although chitosan also has a residue of 35.4%, this value is smaller than in the composite. Finally, in both itaconic acid and the *Randia* extract, residues at 800 °C showed very low residual percentages, which corresponds to an organic compound with volatile thermal decomposition, these parameters are indicated in Table 1.

### 3.6. Cell Viability

Cell viability was performed using the MTT kit by formazan formation. Samples were tested with the healthy cell strain BALB/3T3. In addition to Ch/It and Ch/It/Ran films, the pulverized complete fruit and the *Randia* extract were analyzed. All experimental details can be checked in the corresponding sections of “Materials and Methods”. As additional information, the weight percentage (%wt.) of fractions are shown in Table 3.

Cell viability values obtained in the controls (the medium without the sample) are adjusted to 100% for a proper comparison among samples regarding the effect on cell survival. As expected, Ch/It film was the one that showed the highest cell survival, 84% ± 1.4, since both biomaterials chitosan and itaconic acid are extensively used in the industry [16,47]. The second sample of higher cell survival 77% ± 2.3 was the film with the three components Ch/It/Ran. This slight decrease in cell viability is attributed to the bioactive compounds in the *Randia* extract (22% wt.), even though this sample is also profiled as a biocompatible material since cell survival was sufficient [48]. Lastly, the cell viability percentages for *Randia* complete fruit 12% ± 3.4 and *Randia* extract 9% ± 2.1 were similarly very low. Therefore, it is assumed that 10 mg mL^−1^ exceeds the concentration at which cells can survive in vitro; moreover, both the fruit extract and the complete fruit decrease cell survival in a statistically equivalent percentage, despite the difference in solubility between the extract and the complete fruit (higher solubility and higher amount of bioactive principle in the *Randia* extract). 

**Figure 8:**
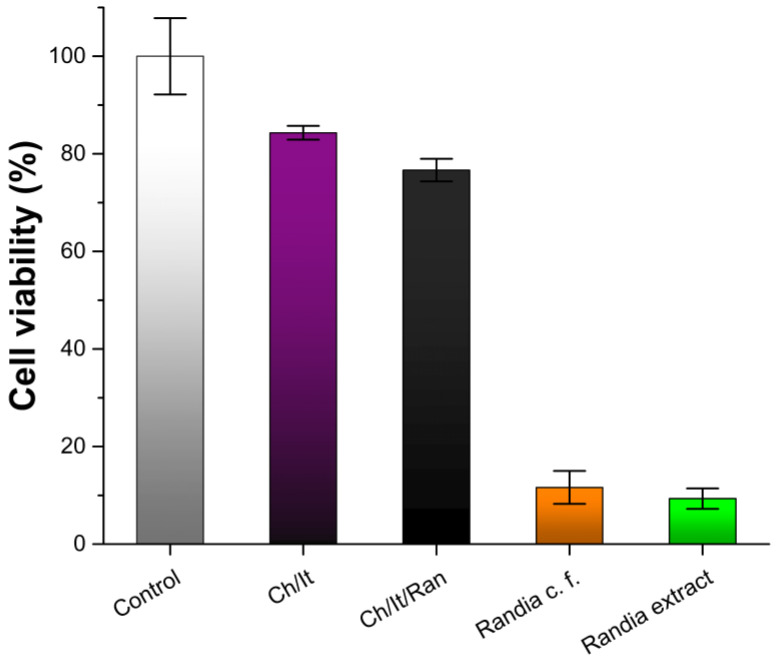
Cell viability (BALB/3T3) of chitosan and itaconic acid film (Ch/It); chitosan, itaconic acid, and *Randia* extract film (Ch/It/Ran); *Randia* complete fruit (*Randia* c. f.); and *Randia* extract.

Based on these preliminary results, it is concluded that the composition of the Ch/It/Ran film produces a hydrogel biocompatible with BALB/3T3, which allows continuing in subsequent stages of analysis (Figure 8). The concentration of bioactive compounds such as flavonoids in the Ch/It/Ran film is acceptable for a biomaterial. As final note, the potential use of this material as a wound dressing can be studied on epithelial regeneration [49], and in the case of satisfactory results, an in vivo study can be proceeded to [50].

## 4. Conclusions

The proposal of ionic crosslinking chitosan provides advantages over other crosslinking methods; firstly, the crosslinking was achieved under reaction conditions free of acetic acid and halogenated crosslinking agents, since itaconic acid enhances crosslinking chitosan, forming a hydrosoluble network. A second benefit is that the reaction system only required water as solvent to form the hydrogel in situ, which is positive from a green approach. Additionally, this methodology can be easily adapted in an industrial process to obtain an interesting material for sanitary dressings because this chitosan-based hydrogel allowed the incorporation of *R. capitata* extract into the network, forming a biomaterial with high cell viability. As a final annotation, we sowed the annals to continue in the study of a Chitosan/Itaconic acid/*Randia* extract system as a biomaterial for medical applications due to the promising bioactive properties of *R. capitata* extract for epithelial wound healing.

## Figures and Tables

**Table 1:** Most important bands of FTIR assigned to starting materials and products [33].

Sample	Wavenumber (cm^−1^)	Type
Chitosan	3354, 3296 (wide band)	O-H st and N-H st
	2868	C-H st
	1575	N-H bd
	1373	C-H (methyl) bd
	1145, 1025	C-O st as and sym
Itaconic acid	3000 (wide band)	O-H st
	3013, 2917	C-H st
	1682	C=O st
	1623	C=C
	1395	C-H bd
	1304, 1213	C-O st
*Randia* extract	3291	O-H st
	2920	C-H st
	1699	C=O st
	1594	C=C
	1403	C-N
	1021	C-O st
Ch/It	3030 (wide band)	O-H st
	2885	C-H st
	1528	C=C
	1375	C-H methyl bd and O-C=O as st
	1066, 1022	C-O st
Ch/It/Ran	3000 (wide band)	O-H st
	2878	C-H st
	1524	C=C
	1375	C-H methyl bd and O-C=O as st
	1151, 1061, 1013	C-O st, as and sym

Abbreviations: stretching (st), bending (bd), asymmetric (as), symmetric (sym).

**Table 2:** Condensed information of DSC and TGA analysis.

Sample	DSC		TGA		
	Transition (°C)	Thermal Transition	10 wt% Loss (°C)	Decomposition Temperature Td (°C)	% Char Yield (800 °C)
Chitosan	101	Endo	275	297	35.4
Itaconic acid	168	Endo	176	203	0.6
*Randia* extract	135,	Endo	176	192, 258	12.5
	191				
Ch/It	128	Endo	224	196, 302	42.1
Ch/It/Ran	170,	Endo	200.3	136, 185,311	54.2
	210				

**Table 3:** Weight percentage of films Chitosan/Itaconic acid and Chitosan/Itaconic acid/*Randia* extract.

	Chitosan (%wt.)	Itaconic Acid (%wt.)	*Randia* Extract (%wt.)
Ch/It	70	30	-
Ch/It/Ran	55	23	22

## Data Availability

The data presented in this study are available on request from the corresponding author.
